# Screening for 15 pathogenic viruses in human cell lines registered at the JCRB Cell Bank: characterization of *in vitro* human cells by viral infection

**DOI:** 10.1098/rsos.172472

**Published:** 2018-05-02

**Authors:** Setsuko Shioda, Fumio Kasai, Ken Watanabe, Kohei Kawakami, Azusa Ohtani, Masashi Iemura, Midori Ozawa, Akemi Arakawa, Noriko Hirayama, Eiko Kawaguchi, Tomoko Tano, Sayaka Miyata, Motonobu Satoh, Norio Shimizu, Arihiro Kohara

**Affiliations:** 1 Japanese Collection of Research Bioresources (JCRB) Cell Bank, Laboratory of Cell Cultures, National Institutes of Biomedical Innovation, Health and Nutrition, Osaka, Japan; 2 Department of Virology, Medical Research Institute, Tokyo Medical and Dental University, Tokyo, Japan

**Keywords:** virus test, cell culture, *in vitro* virus infection, viral tropism, cellular characteristics

## Abstract

Human cell lines have been used in a variety of research fields as an *in vitro* model. These cells are all derived from human tissue samples, thus there is a possibility of virus infection. Virus tests are routinely performed in clinical practice, but are limited in cell lines. In this study, we investigated 15 kinds of viruses in 844 human cell lines registered at the Japanese Collection of Research Bioresources (JCRB) Cell Bank. Our real-time PCR analysis revealed that six viruses, EBV, HTLV-1, HBV, B19V, HHV-6 and HHV-7, were detected in 43 cell lines. Of them, 20 cell lines were transformed by intentional infection *in vitro* with EBV or HTLV-1. Viruses in the other 23 cell lines and one EBV transformed cell line are derived from an *in vivo* infection, including five de novo identifications of EBV, B19V or HHV-7 carriers. Among them, 17 cell lines were established from patients diagnosed with virus-associated diseases. However, the other seven cell lines originated from *in vivo* cells unrelated to disease or cellular tropism. Our approach to screen for a set of 15 viruses in each cell line has worked efficiently to identify these rare cases. Virus tests in cell lines contribute not only to safety assessments but also to investigation of *in vivo* viral infection which can be a characteristic feature of cell lines.

## 1. Introduction

A number of human cell lines have been established from the various tissues of normal individuals, as well as patients with a range of cancers. These resources have been used in a broad research area, including as an experimental model for drug development and to gain a deeper understanding of molecular pathogenesis. General guidelines for the use of cell lines are provided to obtain reliable data from experiments using cultured cells [1,2]. Authentication failures caused by cross-contamination or misidentification and mycoplasma contamination are fundamental issues, which affect the quality of materials in cell culture [3–6]. However, these concerns can be avoided by following the guidelines in accordance with best practices [7,8].

A diverse range of viruses have been identified within specific tissues and reported in relation to disease pathogenesis [9]. Human tissue samples are often subjected to viral infection in diagnostic laboratories and certain viral tests are performed in routine clinical practice. For example, the human herpesvirus family is known as a common pathogen that causes disease in humans, and has been investigated in clinical samples [10]. A method of detecting the herpesviruses has been established based on multiplex real-time PCR, and this assay has been applied in clinical samples, demonstrating a robust screening method [11]. Although human cell lines could potentially carry a virus through an establishment, little attention has been paid to the possibility of viral infections in cell samples.

When viruses enter the human body, they proliferate in susceptible cells and establish an infection. This can cause illness with clinical symptoms such as fever, rash or headache, leading to a certain type of infectious disease unique to infected tissues. Alternatively, viruses are often observed in a latent state, which is sometimes related to reactivation [12]. Viruses are structurally classified by genome types, DNA and RNA, which are represented by herpesviruses and retroviruses, respectively (table 1). DNA viruses retain the ability to repair mismatched base pairs, exhibiting low diversity. RNA viruses are sorted into two groups distinguished by the presence or absence of reverse transcriptase. Because of reverse transcription of the viral RNA into DNA, retroviruses, such as human T-cell leukaemia virus (HTLV) and human immunodeficiency virus (HIV), can be detected in cellular DNA. These RNA viruses are known to be integrated in host cell genome DNA after infection.

**Table 1.** List of pathogenic viruses examined in human cell lines. CMV, cytomegalovirus; EBV, Epstein-Barr virus; HHV-6, human herpesvirus 6; HHV-7, human herpesvirus 7; BKV, human polyomavirus BK; JCV, human polyomavirus JC; ADV, human adenovirus; B19V, human parvovirus B19; HBV, hepatitis B virus; HTLV-1, human T-cell leukaemia virus type 1; HTLV-2, human T-cell leukaemia virus type 2; HIV-1, human immunodeficiency virus 1; HIV-2, human immunodeficiency virus 2; HAV, hepatitis A virus; HCV, hepatitis C virus.

name	genome type	viral tropism	related disease	viral DNA test	viral RNA test
CMV	DNA	monocyte, epithelial cells, endothelial cells	birth defects, cytomegalic inclusion disease	○	
EBV	DNA	B-cell, epithelial cells	infectious mononucleosis, Burkitt lymphoma, nasopharyngeal cancer, CNS lymphoma, leukaemia	○	
HHV-6	DNA	T-cell, NK cell	exanthem subitum (HHV-6B)	○	
HHV-7	DNA	T-cell	exanthem subitum, febrile convulsions	○	
BKV	DNA	reno-urinary tract	haemorrhagic cystitis after allogeneic haematopoietic stem cell transplantation	○	
JCV	DNA	reno-urinary tract, brain	progressive multifocal leucoencephalopathy in HIV-AIDS, haemorrhagic cystitis after allogeneic haematopoietic stem cell transplantation	○	
ADV	DNA	genitourinary tract	haemorrhagic cystitis after allogeneic haematopoietic stem cell transplantation	○	
B19V	DNA	erythroid cells, skin, liver	erythema infectiosum, arthritis, chronic anaemia etc.	○	
HBV	DNA	hepatocyte	hepatitis, cirrhosis and hepatocellular carcinoma	○	
HTLV-1	RNA	T-cell	human T-cell leukaemia	○	○
HTLV-2	RNA	T-cell	human T-cell leukaemia	○	○
HIV-1	RNA	CD4 (+) T-cell	AIDS	○	○
HIV-2	RNA	CD4 (+) T-cell	AIDS	○	○
HAV	RNA	hepatocyte	hepatitis		○
HCV	RNA	hepatocyte	hepatitis, cirrhosis and hepatocellular carcinoma		○

Certain types of viruses are employed to induce cellular oncogenesis and immortalization [13], represented by Epstein–Barr virus (EBV), discovered as the first human oncogenic virus. EBV-transformed lymphoblastoid cell lines are conventionally established from patients with congenital anomalies to provide continuous supplies of genomic DNA as a valuable resource [14]. In contrast to viruses experimentally used for transformation *in vitro*, some viruses originated from *in vivo* infection are detected in certain cell lines. It is reported that HHV-8 was detected in human cell lines specifically derived from primary effusion lymphomas [15]. Screening for seven kinds of viruses has been performed in 460 primate cell lines, revealing that some samples were positive for EBV by PCR but negative by southern blot [16]. This implies that the presence of viruses in cell lines can be more accurately detected by PCR.

Bacterial contamination is visually found in cell cultures through a conventional light microscope, and can be prevented using antibiotics. However, virus detection requires molecular analysis, and, once cells have become infected the viruses cannot be easily removed from the cells. Datasets from virus examination in cell lines contribute to the safe management of cell culture and characterization of cell lines. In this report, we have screened for 15 viruses in 844 human cell lines registered with the Japanese Collection of Research Bioresources (JCRB) Cell Bank and have detected six different viruses in 43 cell lines.

## 2. Material and methods

### 2.1. Cell lines

Cell lines examined in this study are registered with the JCRB Cell Bank and are listed in the electronic supplementary material, table S1. These cells had been authenticated by short tandem repeat (STR) analysis and had been confirmed as mycoplasma-negative in our routine quality control. Certain cell lines carrying the same name appear with different registration numbers. These cell lines were deposited by various depositors through different routes, and would normally have the same characteristics, but there is the possibility of subtle strain differences.

### 2.2. Microbiological tests

The residual cells from every freezing lot were inoculated into two tubes and one agar plate (NB medium—nutrient broth with 2% yeast extract, thioglycollate medium, 1-blood agar plate) for a sterility test. These tubes and plates were incubated at 35°C for 2 weeks to examine bacterial and fungal contamination.

### 2.3. DNA and RNA extraction

Total DNA and RNA samples were extracted from each cell line with the AllPrep DNA/RNA Mini kit (QIAGEN, Germany, 80204). Some DNA was prepared by a conventional protocol using phenol–chloroform. When extracting RNA for copy number titration, DNA contamination was eliminated by digestion with RNase-free DNase I (QIAGEN, Germany, 79254) at room temperature.

### 2.4. Detection of viral DNA with real-time PCR

The viral DNA test was targeted to nine and four kinds of DNA and RNA viruses, respectively (table 1), CMV, EBV, BKV, JCV, HHV-6, HHV-7, ADV, B19V, HBV, HTLV-1, HTLV-2, HIV-1 and HIV-2, and performed using customized 96-well plates inoculated with primers and probes in trehalose solution [17]. This format included two sets of multiplex PCR amplification: one for BKV and JCV, the other for B19V and HBV. Primers and TaqMan probes designed for each virus are shown in the electronic supplementary material, table S2. An aliquot of 50 µl of reaction mixture consisting of 500 ng of the cellular DNA with AmpliTaq Gold DNA Polymerase (Applied BioSystems, USA, N8080245) or Taq DNA Polymerase (Thermo Fisher Scientific, USA, EP0406) was added to each well. The assay was conducted using the Applied Biosystems 7300 Real-Time PCR System, with an initial denaturation at 95°C for 10 min, followed using 50 cycles of 95°C for 15 s and 60°C for 1 min for the AmpliTaq Gold DNA Polymerase or with an initial denaturation at 95°C for 1 min followed by 45 cycles of 95°C for 10 s and 60°C for 30 s for Taq DNA Polymerase.

### 2.5. Detection of viral RNA with One-Step RT-PCR

Viral RNA was examined for six kinds of RNA viruses (table 1), HAV, HCV, HTLV-1, HTLV-2, HIV-1 and HIV-2. The reaction mixture using the One-Step PrimeScript RT-PCR Kit (TaKaRa, Japan, RRO64A) was prepared by adding 100 ng of total RNA following the manufacture's protocol. Primers and TaqMan probes are shown in the electronic supplementary material, table S3. PCR was carried out using the Applied Biosystems 7300 Real-Time PCR System under conditions consisting of 42°C for 5 min and 95°C for 10 s followed by 50 cycles of 95°C for 5 s and 60°C for 34 s.

### 2.6. Reverse Transcriptase Assay

Some cell lines were transfected with the luciferase expressing gene using retrovirus or lentivirus vectors. To examine lentivirus replication, the cultured medium of these cell lines was centrifuged at 250*g* for 10 min at 4°C to remove cell debris. The supernatant was concentrated by centrifugation (22 000*g*) for 2 h at 4°C, then the pellets were analysed by the Reverse Transcriptase Assay (Roche, Germany, 11468120910) as described by the manufacturer.

## 3. Results

Our virus test detected six viruses, EBV, HTLV-1, HBV, B19V, HHV-6 and HHV-7, in 43 cell lines (table 2). One of them, AT(L)7KY, was positive for both EBV and HHV-7. Six cell lines are sublines, corresponding to three individuals, owing to duplicated registrations, different names or misidentification. The other nine viruses were not found in any of the 844 cell lines. Whole results are listed in the electronic supplementary material, table S1 in which positive data are shown by Ct value or DNA copy number. All cell lines were negative for bacterial microorganisms.

**Table 2.** Summary of virus screening in human cell lines.

		*in vivo* infection		
positive virus	number of cell lines	disease associated	disease unrelated	transformed *in vitro*
EBV	30*	12	2	16
HTLV-1	6	1	1	4
HBV	4	4		
B19V	2		2	
HHV-6	1		1	
HHV-7	1*		1	
*double positive	−1			
subtotal	43			
negative	801			
total	844	17	7	20

### 3.1. EBV

The most common virus we detected in human cell lines was EBV, found in 30 cell lines, all of which are derived from blood cells (table 3.1). Two of them, P32/ISH and ITSM, have not been reported to be EBV positive and have been identified in this study. EBV is closely associated with endemic Burkitt lymphoma, but this is low in Japanese cases [18]. Six of nine Burkitt lymphoma cell lines, Daudi, HS-Sultan, Namalwa, NC-37, P32/ISH and RAJI, were positive for EBV in contrast to three negative cell lines, KHM-10B, Minami-2 and Ramos (RA1). NC-37 is a misidentified cell line and has been revealed as the derivative of RAJI. Except for P32/ISH, the other five positive cell lines different from Japanese origin showed a high copy number of EBV. P32/ISH, as described previously [19], has an immature B-cell phenotype on the basis of immunological surface marker analysis. EBV nuclear antigen and terminal deoxynucleotidyl transferase activity were negative, which would be related to low copy number of EBV. The other eight EBV-positive cell lines were established from different haematological diseases, leukaemia, multiple myeloma, lymphoma, pseudomyxoma peritonei or severe chronic active EBV infection. One of them, KAI3, is non-B-cell and has been reported as an NK-like cell line [20]. RPMI 1788 was not associated with any disease and was established from normal cells, which would be EBV positive *in vivo*.

**Table 3.** List of virus-positive cell lines. Bold italic names indicate cell lines first identified as virus positive in this report. Bold italic origins show different tropism compared to available data.

cell name	origin of tissue or cell	JCRB registration no.
1. EBV-positive cell lines		
** **1.1. Burkitt lymphoma		
Daudi	B lymphocyte	JCRB9071
HS-Sultan	B lymphocyte	JCRB9062
Namalwa	B lymphocyte	IFO50040
NC-37^a^	Identical STR profile with the RAJI cell line	IFO50039
***P32/ISH***	B lymphocyte	JCRB0095
RAJI^a^	B lymphocyte	JCRB9012
1.2. leukaemia, myeloma, lymphoma		
CCRF-SB	B lymphoblastoid leukaemia	JCRB0032
IM-9	B lymphoblastoid multiple myeloma	JCRB0024
KAI3	NK-like cell line from a patient with severe chronic active EBV infection	IFO50518
RM-P1	B cell primary effusion lymphoma	JCRB1650
SLVL	B cell lymphoma	JCRB0159
WIL2-NS	B lymphocyte	JCRB9063
1.3. disease-unrelated *in vivo* infection		
***ITSM***	pseudomyxoma peritonei, cecum	JCRB1635
RPMI 1788	peripheral blood from normal individual	JCRB0035
1.4. *in vitro* EBV transformed		
AT(L)5KY	peripheral blood, ataxia telangiectasia patient	JCRB0331
AT(L)6KY	peripheral blood, ataxia telangiectasia patient	JCRB0332
AT(L)7KY	peripheral blood, ataxia telangiectasia patient, HHV-7 positive	JCRB0333
BSL2KA	peripheral blood, Bloom syndrome patient	JCRB1042
GM1526	peripheral blood, ataxia telangiectasia patient	JCRB3008
HLCL-1	peripheral lymphocyte, normal	JCRB0041
KCMC-1528	peripheral blood, Rubinstein–Taybi syndrome patient	JCRB1541
NCC-CoC-K115B	peripheral blood, colon cancer patient	JCRB1339
RB182F(L)	peripheral blood, father of retinoblastoma patient	JCRB3038
RB257F(L)	peripheral blood, father of retinoblastoma patient	JCRB3037
RBL162T	peripheral blood, retinoblastoma patient	JCRB0328
RBL182T	peripheral blood, retinoblastoma patient	JCRB0330
RBL221T	peripheral blood, retinoblastoma patient	JCRB0329
TK6(IVGT)	lymphoblast	JCRB1435
XPL15OS	peripheral blood, xeroderma pigmentosum patient	JCRB0307
XPL3KA	peripheral blood, xeroderma pigmentosum patient	JCRB0306
2. HTLV-1-positive cell lines		
KHM-3S	***small cell lung cancer***	JCRB0138
MT-1	lymphoid cell line from adult T-cell leukaemia, low level HTLV-1 producer	JCRB1209
MT-2	human cord leucocyte cell line established by co-cultivation with human ATL cells	JCRB1210
MT-3	human cord leucocyte cell line established by co-cultivation with human ATL cells	JCRB1217
MT-4^b^	human cord leucocyte cell line established by co-cultivation with human ATL cells	JCRB0135
MT-4^b^	human cord leucocyte cell line established by co-cultivation with human ATL cells	JCRB1216
3. HBV-positive cell lines		
Alexander cells^c^	hepatoma, HBs antigen-positive	IFO50069
huH-1	hepatoma, HBs antigen-positive	JCRB0199
JHH-7	hepatoma	JCRB1031
PLC/PRF/5^c^	hepatoma, HBs antigen-positive	JCRB0406
4. B19V-positive cell lines		
***FPC5JTO***	skin fibroblast of familial polyposis coli patient	JCRB0318
***XP2SA***	skin fibroblast of xeroderma pigmentosum (variant type) patient	JCRB0305
5. HHV-6-positive cell line		
HUV-EC-C	***human vascular endothelial cell***	IFO50271
6. HHV-7-positive cell line		
***AT(L)7KY***	peripheral blood, ataxia telangiectasia patient, EBV transformed	JCRB0333

Six cell lines are sublines consisting of three different *in vivo* origins. ^a^Identical origin because of misidentification in NC-37. ^b^Different registration numbers due to independent depositions. ^c^PLC/PRF/5 has also been used as Alexander cells.

Compared with these 14 EBV-positive cell lines originated from *in vivo* infection, 16 lymphoblastoid cell lines were established from *in vitro* transformation of peripheral blood mononuclear cells using EBV. They were obtained from patients with retinoblastoma and their parents, or patients with a rare disease such as Bloom syndrome, ataxia telangiectasia or xeroderma pigmentosum. Different from these cell lines related to inherited diseases, TK6(IVGT) is characterized by being heterozygous for thymidine kinase and has been used for *in vitro* mutagenesis assays [21]. EBV-transformed cell lines generally proliferate well, and high copy number of EBV was detected.

### 3.2. HTLV-1

HTLV-1 was detected in six cell lines, one lung cancer and four leucocytes (table 3.2). KHM-3S was established from pleural effusion of a patient with small cell lung cancer and showed negative for leucocyte common antigen [22], indicating its unique tropism. MT-1 was derived from a patient with adult T-cell leukaemia (ATL), which shows strong association with HTLV-1 [23]. These two cell lines originated from an *in vivo* infection. MT-2, MT-3 and MT-4 were established by co-culture with lymphoid cells from ATL patients and were infected with HTLV-1 *in vitro* [24]. The viral RNA was detected from these six cell lines and the DNA showed a high copy number. Five types of HTLV-1 proviral sequences were detected in 11 different sites of the MT-2 genome, indicating the integration [25]. It is reported that these MT-1, MT-2, MT-3 and MT-4 cell lines are virus-producing cell lines [26].

### 3.3. HBV

HBV was detected from three hepatoma cell lines, huH-1, JHH-7 and PLC/PRF/5 (table 3.3). PLC/PRF/5 is also known as Alexander cells, registered as IFO50069. The viral DNA copy number varied between these cell lines. It is reported that huH-1 and PLC/PRF/5 carry hepatitis B surface antigen (HBsAg) [27]. Although the target region of our real-time PCR is the S gene coding for HBsAg of the virus envelope, the antigen was not detected in JHH-7 [28]. Two integration sites composed of incomplete HBV genome are identified in the JHH-7 genome [28]. Other hepatoma cell lines without HBsAg, JHH-1, -2, -4, -5 and -6, were negative for HBV [28], indicating that JHH-7 exhibits a distinctive feature in the HBV-positive cells.

### 3.4. B19V

B19V was detected in two cell lines, FPC5JTO and XP2SA (table 3.4), which could be the first cell lines positive for B19V, originated from *in vivo* infection. These cell lines were fibroblast derived from the skin of patients with hereditary diseases, carrying a high cancer risk. Different from the EBV, HTLV-1 and HBV detected mostly in cancer cell lines, the two B19V-positive cell lines have no association with diseases. Their copy numbers were low, which would be related to their primary cells. Although B19V can propagate in a leukaemia cell line, UT-7 [29], the positive cell lines have not been registered with any major public cell banks.

### 3.5. HHV-6

HHV-6 was detected in a human vascular endothelial cell line, HUV-EC-C, derived from normal umbilical cord tissue (table 3.5). We have previously reported that chromosomally integrated HHV-6B has been identified at the distal end of chromosome 9q in this particular cell line [30]. The copy number is calculated to be one copy per cell, indicating that the integration could have occurred in the germ line. The viral integration in the HUV-EC-C cell line would be caused by *in vivo* infection. This is distinct from other HHV-6-positive cell lines generated by *in vitro* transfection [31].

### 3.6. HHV-7

HHV-7 was detected in one cell line, AT(L)7KY, which was not previously reported to be positive. This cell line is a B-cell line transformed by EBV, indicating that both EBV and HHV-7 can be detected in this cell line (table 3.6). It was established from peripheral blood mononuclear cells of a patient with ataxia telangiectasia, which is not associated with HHV-7 infection. Different from HHV-7-infected cell lines transduced with an adenovirus vector [32], AT(L)7KY is, to the best of our knowledge, the first report of an HHV-7-positive cell line likely to be originated from *in vivo* infection which remains detectable in cell culture.

### 3.7. HIV-1 and HIV-2

A very small copy number of DNA fragments targeting the gag gene of HIV-1 and LTR of HIV-2 were detected in some of the luciferase-expressing cell lines (electronic supplementary material, table S4). However, these viral RNAs were not detected in these cell lines, and reverse transcriptase activity was below the detection limit. The absence of the RNA and reverse transcriptase in these cell lines confirmed that the cell lines are negative for HIV-1 and HIV-2. The luciferase gene was introduced into these cell lines by the lentiviral vector [33], which may be associated with the detection by PCR. Because HIV-1 and HIV-2 are highly pathogenic and must be handled under biosafety level 3 (BSL3), such cell lines are not received and distributed from our JCRB Cell Bank. All cell lines examined in this study are free of HIV-1 and HIV-2, which minimizes safety concerns over the use of our cell lines.

## 4. Discussion

Virus infection is one of the major public health concerns and is related to disease pathogenesis [34]. Virus tests have been established for clinical samples in diagnostic laboratories [9–11]. These tests are also routinely performed on donated blood to avoid infections through blood products. Blood samples are examined mainly by targeting antigens or antibodies detected in plasma. Positive results from these indirect methods identify individuals carrying a viral infection, but do not specify which cells carry the virus. Different from those on *in vivo* samples, our virus tests for cell lines detect viral DNA or RNA in the cytoplasm or the nuclei. The presence of these viral components in cells can be a molecular characterization of the cell line.

Our previous study demonstrated that chromosomally integrated HHV-6B has been identified in a human vascular endothelial cell line, HUV-EC-C (IFO50271) [15]. The analysis of the HUV-EC-C cells reveals that integration of HHV-6B is stable during long-term culture of HUV-EC-C, despite genomic instability in the host cell. A complete HHV-6 genome can be present as the form of integration into the host germ line genome [35], leading to the unexpected detection in different cell types of tropism observed as the HUV-EC-C cell line. It was reported that a human B cell line, RPMI 1788, was established from the peripheral blood of a normal donor and labelled as normal cells [36], however this cell line was found to be EBV positive in another study [37]. This suggests that lymphocytes had been infected with EBV *in vivo*, leading to establishment of the immortalized cell line. These examples show that further investigation of virus-positive cell lines could lead to an understanding of viral dynamics and that such cell lines can serve as invaluable research materials. Virus tests contribute to the characterization of cell lines and elucidation of cellular origins. Because each cell line is derived from a specific tissue and established from a certain cell population in the sample, cell lines that show negative on virus tests are not necessarily derived from uninfected individuals.

Cells *in vitro* always have a risk of viral infection in the culture environment because, by themselves, they lack a defence system against infection. Assessment of virus contamination in cells is undertaken for the purpose of risk management [38]. When cell lines are used as biological products in the biotechnology industry, quality control is strictly maintained during production [39]. Examination of the parainfluenza virus has been performed in 123 human cell lines without any such infection being detected, assuring that those lines are free from the infection [40]. Another study of a screen for murine leukaemia viruses in 577 human cell lines reported that 17 of these cell lines produced active retroviruses [41]. This implies that human cell lines have a risk of viral contamination through transplantation of cells into mice, which is distinguished from *in vivo* infection characterizing cell lines. The use of human cell lines is accompanied by a potential biohazard, because virus tests are limited to certain species and do not cover all pathogens. It is recommended that human cell lines are primarily handled at a biosafety level 2 laboratory, regardless of results from virus tests. Especially, cell lines which produce virus particles need careful handling and their application should be restricted. Each virus has distinct host cell tropisms, implying that negative results would be expected from the majority of cell lines. Because it is not clear that viral tropism is identical between *in vivo* and *in vitro* environments, we applied the whole set of virus tests to all cell types. Apart from the cell lines transformed using EBV or HTLV-1 by *in vitro* infection, 22 cell lines had been infected with virus *in vivo* before their establishment. Of them, two cell lines are inconsistent with the cellular tropism, showing unexpected results.

It is reported that induced pluripotent stem cells can be established from EBV-transformed lymphoblastoid cell lines, and that the reprogrammed cells have been shown to be EBV free [42,43]. This suggests that a change in cell type could lead to elimination of viruses infecting cell lines. These viruses might present in the form of episomes in cells, which are different from integration into host genomes. Differences in the form of viruses in cells would be related to the amount of virus present during cell culture. In this report, human papillomavirus (HPV) is excluded because it has diverse genotypes related to malignant potential, which we will analyse in a future study. Analyses of RNA sequencing data led to identification of EBV, HHV-8 and HTLV-1 in lymphoma cell lines [44]. DNA sequence data from a large number of human cancer cell lines have been accumulated in the Cancer Cell Line Encyclopedia (CCLE) [45] and the Catalogue of Somatic Mutations in Cancer (COSMIC) [46]. Virus-positive cell lines will be identified from these databases, and bioinformatics approaches will help to investigate viral integration sites and genotypes.

## Supplementary Material

List of all cell lines screened for 15 viruses

## Supplementary Material

Primers used for viral DNA test by real-time PCR; Primers used for viral RNA test by real-time PCR; HIV-1/HIV-2 positive cell lines

## Data accessibility

The datasets supporting this article have been uploaded as the electronic supplementary tables.
