# Supplementary material for: Screening for 15 pathogenic viruses in human cell lines registered at the JCRB Cell Bank: characterization of in vitro human cells by viral infection
Source: R Soc Open Sci. 2018 May 2;5(5):172472. doi: 10.1098/rsos.172472 (PMC5990783; doi:10.1098/rsos.172472)
Supplement: Primers used for viral DNA test by real-time PCR; Primers used for viral RNA test by real-time PCR; HIV-1/HIV-2 positive cell lines [file rsos172472supp2.pdf]

Supplementary Table 2. Primers used for viral DNA test by real-time PCR

Virus name	Type (DNA or RNA)	Target rigion	Primer sequence (F)	Primer sequence (R)	Probe sequence	product size
GAPDH	PCR control		tgtgctcccactcctgatttc	cctagtcccagggctttgatt	FAM-aaaagagctaggaaggacaggcaacttggc-iowaBlack	76 bp
CMV	DNA virus	Major immediate-early region	catgaaggtctttgcccagtac	ggccaaagtgtaggctacaatag	FAM-tggcccgtaggtcatccacactagg-TAMRA	130 bp
EBV	DNA virus	BALF5 gene	cggaagccctctggacttc	ccctgtttatccgatggaatg	FAM-tgtacacgcacgagaaatgcgcc-iowaBlack	220 bp
HHV-6	DNA virus	U66/65 gene	gacaatcacatgcctggataatg	tgtaagcgtgtggtaatggactaa	FAM-agcagctggcgaaaagtgctgtgc-iowaBlack	176 bp
HHV-7	DNA virus	U37 gene	cggaagtcactggagtaatgacaa	ccaatccttccgaaaccgat	FAM-ctcgcagattgcttgttffccatg-TAMRA	126 bp
BKV	DNA virus	Large T antigen	ggaaagtctttagggtcttctaccttt	gatgaagatttattYtgccatgaRg	FAM-atcactggcaaacat-MGB	119 bp
JCV	DNA virus	Large T antigen		gaagacctgttttgccatgaaga		113 bp
ADV*	DNA virus	Hexon gene	gacatgacttttgaggtgga	tcgatgacgccgcggtg	FAM-cccatggaYgagcccaccct-TAMRA	105 bp
B19V	DNA virus	VP1 protein gene	gggtttcaagcacaagYagtaaaaga	cggYaaacttccttgaaaatg	FAM-cagctgcccctgtgg-MGB	84 bp
HBV	DNA virus	S gene	gtggtggacttctctcaattttctag	ggacaMacgggcaacatacct	FAM-tgtctgcggcgtttt-MGB	222 bp
HTLV-1,-2	RNA virus	Env gene	SHTF : ggccacctgtccagagca	R1 : ctgagccgataacgcgtcca<br>R2 : ctgagctgacaacgcgtcca	FAM-Mtcacctgggaccccatcgatgga-TAMRA	62 bp<br>60 bp
HIV-1	RNA virus	gag gene	F1 : ggacatcaagcagcYatgcaaatg<br>F2 : ggacaccaRgcagctatgcaaatg	R1 : tgctatRtcacttccccttggttctct<br>R2 : tgctatatcacttcccctaggttccct<br>R3 : tgctatatcactcccccctaggttctct	FAM-acHatcaatgaggaagctgcagaa-MGB	135 bp
HIV-2	RNA virus	LTR	gcaggtagagcctgggtgttc	cttgcttctaaYtggcagctttatt	FAM-tgggcagaYggctccacgc-TAMRA	119 bp

*ADV primers target for subtypes 1, 2, 3, 5, 6, 7, 8, 10, 11, 12, 16, 17, 19, 21, 28, 31, 34, 40 and 48.

Supplementary Table 3. Primers used for viral RNA test by real-time PCR

Virus name	Type (DNA or RNA)	Target region	Primer sequence (F)	Primer sequence (R)	Probe sequence	Product size
β-actin	PCR control		cttccttcctgggcat	tcttcattgtgctgggt	FAM- tccgcaaagacctgtacgccaacac -iowaBk	189 bp
HAV	RNA virus	5`UTR	Rggtaggctacgggtgaaacc	gccgctgttaccctatccaa	FAM- tacttctatgaagagatgc -MGB	71 bp
HCV	RNA virus	5`UTR	gtctagccatggcgttagta	ctcgcaagcaccctatcaggcagt	FAM- tgcggaaccggttgagt -MGB	235 bp
HTLV-1,-2	RNA virus	Env gene	SHTF : ggccacctgtccagagca	R1: ctgagccgataacgcgtcca<br>R2: ctgagctgacaacgcgtcca	FAM-Mtcacctgggaccccatcgatgga-TAMRA	62 bp<br>60 bp
HIV-1	RNA virus	gag gene	F1 : ggacatcaagcagcYatgcaaatg<br>F2 : ggacaccaRgcagctatgcaaatg	R1 : tgctatRtcacttccccttggttctct<br>R2 : tgctatatcacttcccctaggttccct<br>R3 : tgctatatcactccccctaggttctct	FAM-acHatcaatgaggaagctgcagaa-MGB	135 bp
HIV-2	RNA virus	LTR	gcaggtagagcctgggtgttc	cttgcttctaaYtggcagctttatt	FAM-tgggcagaYggctccacgc-TAMRA	119 bp

Supplementary Table 4. HIV-1/HIV-2 positive cell line: all luciferase stably expressing cell lines

Cell No.	Cell Name	HIV-1/DNA	HIV-2/DNA	Parent cell line: characteristics
JCRB1454	AsPC-1/CMV-Luc	+	-	AsPC-1: pancreatic adenocarcinoma cell line
JCRB1438	BT-20/CMV-Luc	+	-	BT-20: breast cancer cell line
JCRB1450	BT-474/CMV-Luc	+	-	BT-474: breast cancer cell line
JCRB1683	HARA CMV-Luc	+	+	HARA: lung squamous cell carcinoma cell line
JCRB1508	HCC-1937/CMV-Luc	+	-	HCC1937: breast cancer cell line
JCRB1679	HeLa/CMV-Luc	+	+	HeLa; cervical carcinoma cell line
JCRB1592	HepG2-Luc	+	+	Hep G2: hepatoma cell line
JCRB1670	HT-1080/CMV-Luc	+	+	HT-1080: sarcoma cell line
JCRB1600	HuH-7-Luc	+	+	HuH-7: hepatoma cell line
JCRB1485	IM95/CMV-Luc	+	-	IM95: gastric cancer cell line
JCRB1579	Ishikawa3-H-12-Luc	+	+	Ishikawa3-H-12: endometrium cancer cell line
JCRB1559	MDA-MB-231-Luc	+	+	MDA-MB-231: breast cancer cell line
JCRB1681	MIAPaCa-2/CMV-Luc	+	+	MIA PaCa-2; pancreatic cancer cell line
JCRB1433	MKN-1/CMV-Luc	+	-	MKN1: stomach, adenosquamous carcinoma cell line
JCRB1473	MKN-74/CMV-Luc	+	-	MKN74: stomach, adenocarcinoma cell line
JCRB1609	OVCAR-3/CMV-Luc	+	-	OVCAR-3: ovarian cancer cell line
JCRB1558	RERF-LC-KJ/CMV-Luc	+	-	RERF-LC-KJ: lung cancer cell line
JCRB1627.1	SK-BR-3-Luc	+	+	SK-BR-3: breast cancer cell line
JCRB1594	SK-OV-3/CMV-Luc	+	-	SK-OV-3: ovarian cancer cell line
